# Effect of different crystalline directions on the mechanical properties and processing maps of Zr-Nb alloy for orthopedic applications

**DOI:** 10.1016/j.heliyon.2025.e41834

**Published:** 2025-01-09

**Authors:** Ali Rajaee, Mohsen Asadi Asadabad, Behrooz Shayegh Boroujeny

**Affiliations:** a Department of Materials Engineering, Faculty of Engineering, Hakim Sabzevari University, Sabzevar, Iran; b Nuclear Science and Technology Research Institute, Tehran, Iran; c Materials Engineering Department, Faculty of Engineering, Shahrekord University, Shahrekord, Iran

**Keywords:** Zr alloys, Microstructure, Processing maps, Mechanical properties, Constitutive equations

## Abstract

In the current study, the effect of direction on the hot compression behavior, processing map, and microstructure of Zr-Nb alloy after bidirectional hot forging was studied. Accordingly, the alloy billet was hot-forged and samples at different directions were extracted. Phase changes and microstructure of the samples were characterized. Additionally, hot compression tests were carried out on the samples in the temperature and strain rate ranges of 800–900 °C and 0.001–1 s^−1^, respectively. Microstructure examinations revealed that the forging resulted in the formation of various alpha spherical grains elongated along the forging direction. Along the directions of 30 and 60°, the high strain rate during forging caused the formation of secondary recrystallized grains in addition to grains elongated in the forging direction. Hardness measurement results showed that the highest hardness was related to the zero-degree direction due to the high fraction, refinement, and morphology of the alpha phase formed during the hot forging process. Full recrystallization of hot-compression samples was evident at 850 °C. Processing maps suggested the optimum deformation of the alloy to be within the strain rates 0.01–0.001 at 850 °C. Consequently, deformation within this range results in the desired dynamic recrystallization phenomenon.

## 1 Introduction

Different metallic alloys e.g. Ti alloys, stainless steel grades, Zr alloys, and Co-Cr alloys are employed for orthopedic implant applications. Among them, Zr alloys are considered more desirable for medical applications, since their lower elastic modulus is close to that of the human bone in addition to high biocompatibility and excellent mechanical properties [[1], [2], [3]]. Furthermore, the magnetic susceptibility of Zr alloys is much lower than other metallic implant materials, which highly facilitates the diagnostic technique of resonance imaging (MRI), when needed [4,5]. Hence, low-magnetic-susceptibility materials should be selected for orthopedic implants to avoid artificial image generation, heat induction, and implant displacement during the MRI. Zirconium alloys, especially Zr-Nb series with high omega (ω) phase fractions display the lowest magnetic susceptibility among other biocompatible metals [[6], [7], [8]]. Therefore, the Zr-Nb series are considered perfect candidates for dental implants, knee joints, hip joints, and pedicle screws. Nevertheless, the mechanical behavior and deformation characteristics of these alloys should be studied using processing maps to improve their mechanical properties and manufacturability.

Previous investigations into Zr-Nb alloy properties have revealed an alpha-matrix microstructure containing distributed β-precipitates (with BCC crystalline structure). Because of the restricted mobility of substitutional Nb atoms in the alpha matrix (with HCP crystalline structure), the alloys are in a non-equilibrium state, which means the supersaturation of the matrix with Nb atoms [9,10]. Rare Laves-phase precipitates including Nb atoms are typically evident in the alloys due to the presence of trace iron or chromium atoms [11]. The detected Laves phases are usually amorphized and dissolved during industrial operation. Generally, the formation of refined beta phases reduces the content of Nb within the Zr-Nb alpha matrix. The mechanical properties, magnetic susceptibility, and biocorrosion behavior are extremely reliant on the microstructure (solute atoms and precipitates in the alpha matrix), it is important to perceive the thermodynamics of the Zr-Nb system as well as the interface mechanisms of alpha and beta phases [12].

Experimental data for the Zr-Nb phase diagram have indicated the presence of a non-solubility region, leading to areas with no solubility limit, which are rich in Zr and Nb at absolute zero. According to calculations of CALPHAD software by Guillermet [13], the solubility of Zr in the beta phase at 890 °C (monotectoid transformation) is near 8 atomic percent. At the same time, the solubility of Nb in the alpha phase is considerably lower i.e. 0.8 atomic percent. Nevertheless, there is a great discrepancy in the previous studies concerning the phase boundaries of the Zr-Nb phase diagram. It should be noted that reaching an equilibrium is challenging because of the low diffusion of Nb in Zr and the much lower diffusion of Zr in Nb at low temperatures. In addition, the presence of impurities highly affects these solubility limits, especially the interstitial atoms such as oxygen and substitutional alloy elements such as iron and chromium. As a result, the calculated values for solubility limits at the monotectoid temperature range from 6 to 15 at% Zr in the beta phase and from 0.5 to 1.5 at% Nb in the alpha phase [14]. However, Guillermet's phase diagram [13] is preferred because it has inherent thermodynamic compatibility with the CALPHAD method. These solubility limits were calculated indirectly based on the results of laboratory data in cases where impurities were tightly lowered during extended annealing periods [15,16]. The solubility limit at the optimal temperature range was mainly investigated by the X-ray diffraction (XRD) analysis conducted by Flewitt et al. [15] in the Nb-rich region and the data collected via XRD, resistivity, microscopic evaluation, and dilatometry examinations by Van Effenterre [16] in the Zr-rich region.

The deformation mechanism of zirconium is limited due to the anisotropic crystallographic structure of the HCP system [17]. A recent investigation showed that the initiation of various twinning and slip systems relies on the angle between the basal plane and the loading direction (X_B_). It was reported that at liquid nitrogen temperature, when X_B_ < 35°, deformation is caused by the first-order prismatic slip, and when X_B_ > 35°, {11–21} twinning and prismatic slip are the dominant deformation mechanisms [18]. In another study [19] it was reported that pure Zr under compression perpendicular or along the <c> axis showed different strain hardening behaviors due to activation of twinning or slip systems.

All in all, the energy absorbed during the metal deformation is only around 5–10 % of the total, while the rest is stored by the dislocation structure (generation and movement). Therefore, the amount of stored energy is dependent on the direction. Moreover, it highly affects the crystallization phenomenon (i.e. grain nucleation and growth), influencing the grain texture and microstructure evolution. It was determined that if all the slip mechanisms (i.e. prismatic plane, basal plane, and <c + a> pyramidal) were triggered, the discontinuous dynamic recrystallization (DDRX) was initiated more easily in the HCP structure [20,21].

It has been shown that the mechanical properties of Zr alloys are affected by the primary texture. Likewise, it has been reported that a high fraction of the recrystallized grains form with low internal misorientation when the loading direction is perpendicular to the <c> axis [22]. Several investigations reported characteristics of dynamic recrystallization (DRX) for the alloys. These investigations described different types of DRX phenomena namely DDRX, geometric dynamic recrystallization (GDRX), and continuous dynamic recrystallization (CDRX) [[23], [24], [25]]. They stated that deformation mechanisms are highly dependent on DRX mechanisms.

The dominant mechanism during hot working primarily affects the microstructure. Different phenomena e.g. dislocation generation, dislocation cross slip and glide, diffusion, and climb control the dominating mechanism. Numerous restoration mechanisms were reported for Zr-alloys, comprising CDRX, GDRX, DDRX, and dynamic recovery (DRV) along with grain growth [26].

Parameters of temperature, strain rate, and strain considerably influence the deformation performance, recrystallization phenomena, and phase transformation [27]. Numerous investigations on Zr-based alloys have evaluated the role of these parameters. In a study, Tan et al. [28] studied the effect of temperature and strain rate on the alpha-to-beta transformation for 47Zr−45Ti−5Al−3V alloy. They stated that the fraction of the globular alpha phase is increased by elevating the temperature and reducing the strain rate. In another investigation, Saxena et al. [29] constructed the power dissipation maps of Zr-2.5Nb and evaluated the deformation behavior. They reported two optimal DRX regions at 750 °C and 0.01 s^−1^ and 925 °C and 0.01 s^−1^ with efficiency values of 54–60 % and 49–54 %, respectively. Conversely, they detected an instability range at ∼725 °C and 0.1 s^−1^. Saxena et al. [30] studied the hot deformation behavior of Zr-1Nb alloys in the two-phase region. They reported the highest yield for the sample hot-deformed at 815 °C and 1 s^−1^. They stated that this could be due to the formation of refined alpha lath and high dislocation density. Sarkar et al. [31] showed that the DRX phenomenon resulted in the formation of new grains between the deformed grains for Zr-1Nb alloy samples with high m values.

Consequently, a vast study is needed to investigate these relations in more detail. To this aim, Zr-1Nb alloy samples at four different crystalline directions were hot-compressed in the temperature and strain rate ranges of 800–900 °C and 0.001–1 s^−1^, respectively. Afterward, constitutive equations were employed to analyze the obtained hot compression data and to construct the processing maps for each direction.

## 2 Materials and methods

### 2.1 Alloy preparation

Commercially pure zirconium sponges and niobium plates were melted via vacuum arc melting to prepare the Zr-1Nb alloy ingot. Then, vacuum arc remelting was employed to homogenize the alloy composition. The melt was poured into a cylindrical copper mold with a height of 120 cm and a diameter of 35 cm. The oxygen content was controlled to be maintained under 1200 ppm. Table 1 presents the chemical composition of the prepared alloy.

**Table 1:** chemical composition of the Zr-Nb alloy.

Element	N	Al	Be	B	Hf	Fe	Cd	Ca	P	Si	Li	Mg	Cu
wt%	0.002	0.004	0.001	0.00005	0.004	0.017	0.00002	0.003	0.002	0.002	0.0001	0.001	0.001

Element	Mo	Ni	Pb	Sn	Ti	C	Cl	Cr	H	F	O	Nb	Zr

wt%	0.004	0.002	0.002	0.02	0.002	0.0150	0.002	0.0048	0.001	0.0015	0.089	1.01	Ball.

### 2.2 Bidirectional free forging process

**Fig. 1:**
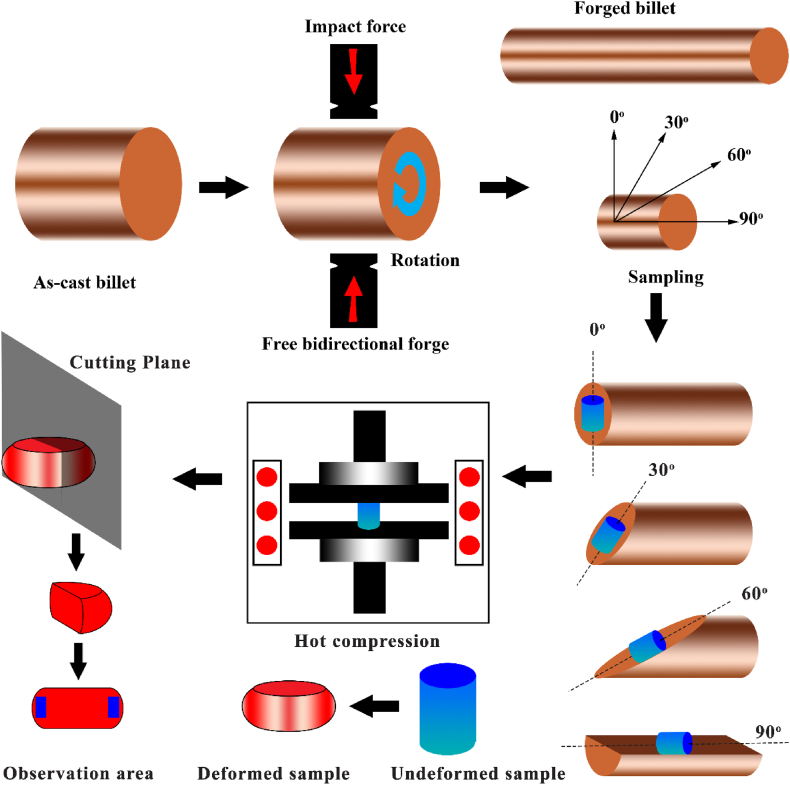
Schematic of the forging process and sampling for hot compression behavior.

A hot bidirectional forging process was carried out on the alloy to induce a primary crystalline orientation. Before the process, MoS_2_ powder was applied on the surface of the ingot as a lubricant. The billet was forged six passes according to the setup in Fig. 1. The billet was reheated to reach the temperature of the alpha-beta region before each pass for 30 min. During each pass, the diameter reduction was 15 %. The actual strain was calculated via ε = lnH/h (where h and H are the ingot diameter after and before each pass, respectively). In this case, a total strain of 1.07 was achieved after six forging passes. After the process, it was cooled in still air.

### 2.3 Mechanical properties

The compression tests were performed using a ZwickRoell universal testing machine (model Z250, Germany) at 800, 850, and 900 °C under strain rates of 0.001, 0.01, 0.1, and 1 s^−1^ on the as-forged samples in 4 directions of 0, 30, 60, and 90° concerning the forging direction. Compression test specimens were prepared with a height of 10 mm and a diameter of 5 mm as per ASTM E9/E9M standard. To reduce the friction effect during the hot compression tests, graphite powder was applied on the contact surfaces of the samples. Furthermore, to validate the obtained data, the compression tests were replicated three times. The reproducibility results showed that the error range of the acquired data was lower than 5 % for all tests. However, the average values were reported. The hardness test was performed by a Vickers hardness tester with a load of 10 kgf for 10 s. It should be noted that each sample was subjected to hardness measurement five times and the average measured values were reported along with the error bars.

### 2.4 Microstructural characterization

The samples were ground by 100–1500 grit sandpapers, polished by diamond paste, and etched by a composition of 8.5 mL HNO_3_, 1.5 mL HF, and 90 mL ethanol. The sample microstructures were attained by Olympus optical microscope (model GX51, Japan) and TESCAN scanning electron microscope (SEM) (model Vega 3, Czech Republic). For phase characterization, XRD analysis was carried out by Philips XRD (model PW1800, Netherlands) at a working voltage and current of 30 kV and 30 mA, respectively, using the CuKα radiation (λ = 1.54184 Å).

## 3 Results and discussion

### 3.1 Evaluation of as-cast samples

**Fig. 2:**
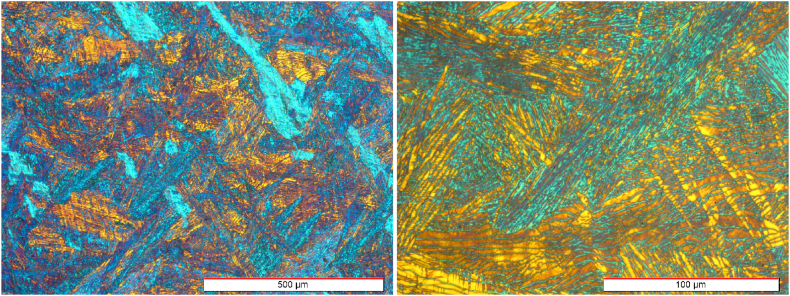
OM micrographs of the as-cast sample.

Fig. 2 shows the microstructure of the as-cast Zr-1Nb sample at different magnifications. Various alpha-phase colonies as well as allotriomorphic alpha grains along the beta grain boundaries and inside beta grains are evident in the microstructure. A few ultrafine secondary alpha phases can also be seen within the beta matrix. The higher magnification micrograph (Fig. 2) demonstrates martensite laths and regions rich in niobium, which are formed due to high cooling rates. Moreover, the as-cast sample microstructure shows coarse coaxial grains in the scale of a few millimeters.

### 3.2 Microstructure of forged samples

**Fig. 3:**
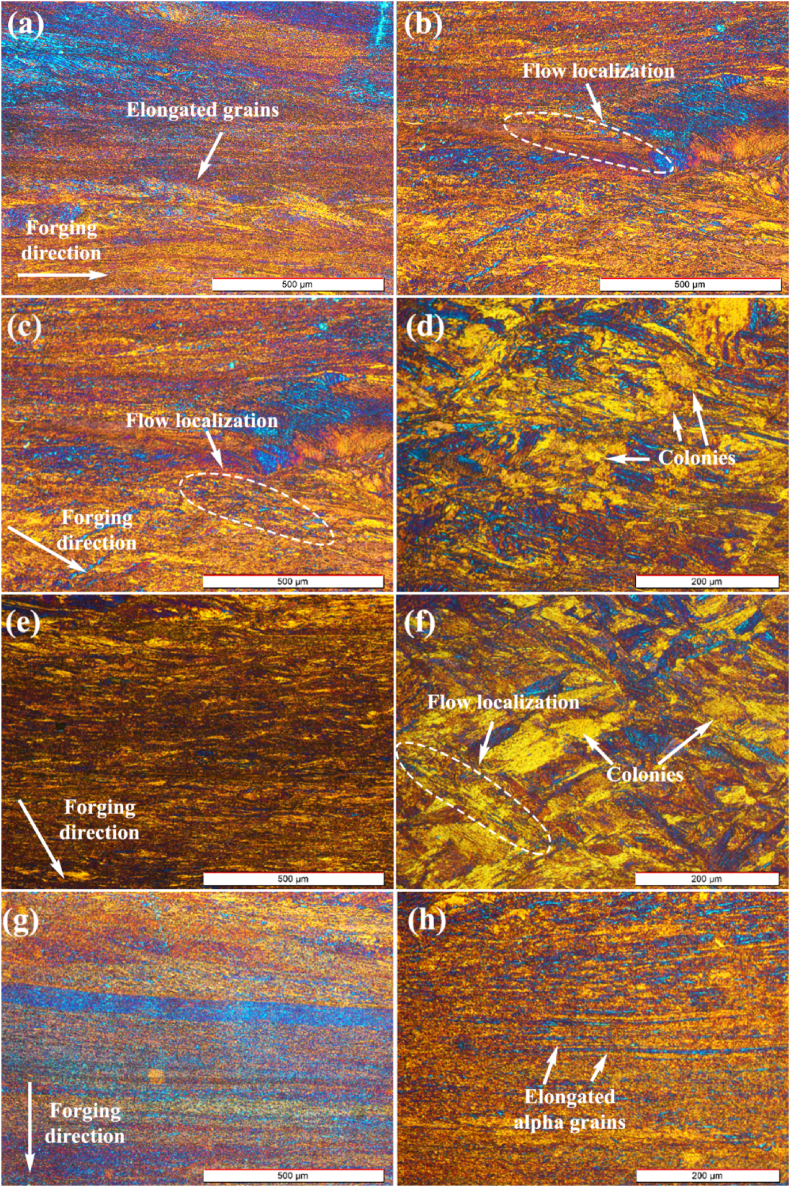
OM micrographs of the forged samples in different directions: (a and b) 0°, (c and d) 30°, (e and f) 60°, and (g and h) 90°.

Fig. 3a and b shows the microstructure of the as-forged sample at zero-degree. As can be seen, forging resulted in the formation of various α grains elongated in the forging direction in the form of fiber. Moreover, flow localization is apparent in the as-forged microstructure because of low deformation temperature, insufficient deformation time, and severe plastic deformation. The work-hardening effect is visible during the deformation process, encouraging the flow localization. This is caused by a severe non-uniform plastic deformation [32]. While for sample 30-degree, the fraction of the elongated α grains has decreased and the size of the colonies has increased. Severe deformation and high cooling rates have controlled the microstructure [33]. In the micrograph with higher magnification, the uneven size distribution of the laths can be seen in the microstructure, mostly due to the alloy phase transformation during rapid cooling (Fig. 3c and d). For sample 60-degree, the decrease in the volume fraction of the stretched alpha grains is less than that of sample 30-degree, and larger colonies can be observed (Fig. 3e and f).

The microstructure of sample 90-degree contains the highest fraction of the elongated α grains (Fig. 3g and h). It can be said that the enhanced strain rate during the forging caused the formation of new secondary α grains. Also, refined secondary alpha laths can be observed between primary alpha grain boundaries during thickness reduction. A similar microstructure was reported for Zr-2.5Nb alloy after hot-rolling and cooling in the air [34]. In general, it can be said that the hot forging characteristics of the alloy are similar to those of hot rolling for Zr-Nb and Zr-Ti binary alloys, observing beta phase in the primary alpha grains.

### 3.3 XRD

**Fig. 4:**
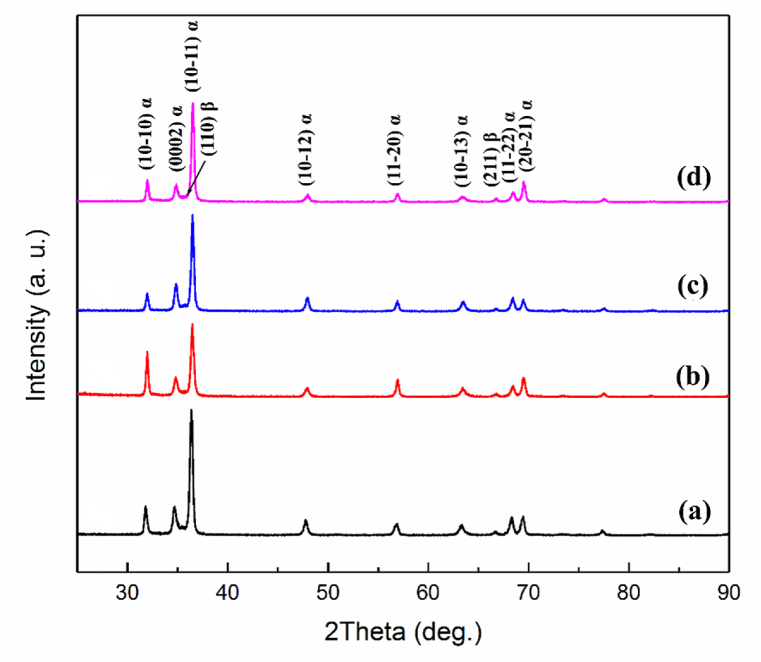
XRD patterns of the forge sample at: (a) 0, (b) 30, (c) 60, and (d) 90-degree directions.

XRD patterns of as-forged samples in different directions are brought in Fig. 4. As expected, Zr and Zr-Nb phases were identified in all directions. Peaks related to the HCP structure were detected in the alloy. Samples show low-intensity BCC (β-Zr as the minor phase) and high-intensity HCP α-Zr (as the major phase). Among the peaks, α (10–11) planes have the highest diffraction intensity. Hence, the HCP structure peaks reveal the presence of α, assuming the transformed structure of the Zr-Nb alloy. By comparing the XRD patterns in different directions, it is clear that the intensity of the peaks is different. This indicates that the deformation results in the generation of different amounts of dislocations in various directions.

### 3.4 Hardness

**Fig. 5:**
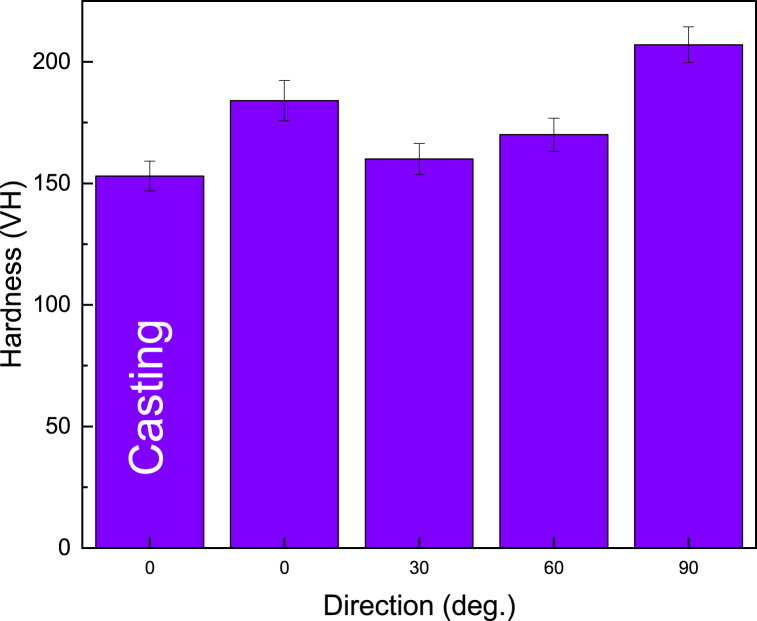
Hardness variation of the as-cast and forged sample at 0, 30, 60, and 90-degree directions.

In Fig. 5, the hardness measurement results for as-cast and forged samples in the directions of 0, 30, 60, and 90° are presented. The hardness of the as-cast sample was 153 HV, while values of 184, 160, 171, and 207 HV were measured for samples forged at 0, 30, 60, and 90°, respectively. By comparing the hardness of the as-cast sample to that of the forged samples, it is evident that the hardness of forged samples increased significantly. Noticeably, severe plastic deformation induced by the forging process results in a rise in hardness by dislocation pile-ups. The measured hardness results were consistent with previous investigations [[35], [36], [37]]. As can be seen, the highest hardness was obtained for sample 90-degree. By comparing the microstructures of the forged sample at different angles, it is evident that the grains are refined during the forging process. Different microstructures observed in different directions indicate various conditions of dislocation motion and obstacles. Additionally, the variation of the volume percentage of the alpha phase and its morphology (spherical or elongated) can change the hardness of the alloy. Results displayed that the microstructure was refined for sample zero-degree, while the microstructures of samples 30-degree and 60-degree were coarsened. Additionally, the microstructure of sample 90-degree showed recrystallized grains.

### 3.5 High-temperature compression test

**Fig. 6:**
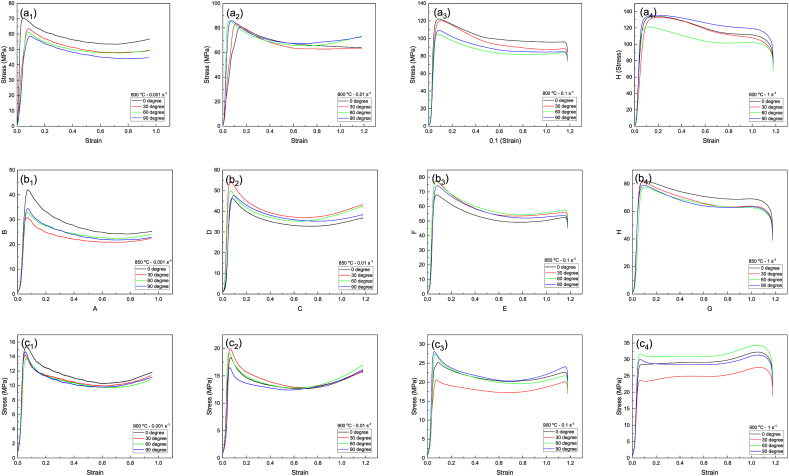
Compressive stress-strain diagrams obtained at: (a) 800 °C, (b) 850 °C, (c) 900 °C and different strain rates in different forging directions. All curves related to sample zero-degree were reprinted from Ref. [42] with permission from Springer.

Fig. 6a–c demonstrate the stress-strain diagrams of samples in different forging directions at 800, 850, and 900 °C and various strain rates. As can be seen, the flow stress increases linearly following the generation of tensile twinning during the first stages of deformation [38]. The flow curves typically indicate work-hardening (flow stress enhancement) up to a strain of 0.1 for all strain rates. Afterward, the flow stress reduces with a mild slope, caused by dynamic softening [39]. Once the strain rate increases to 1 s^−1^ at 900 °C, the changes in flow stress values for all samples in different directions remain constant until the strain value of 1. In the next stage, when work hardening is limited, the curves enter a softening region. According to the figure, after the softening stage, the slope of the curve reduces to near zero, which is caused by a dynamic balance of the flow. This is related to the DRX phenomenon during hot compression [40]. When the graphs with various strain rates are compared together, it is evident that the flow stress declines with increasing temperature.

The microstructure shows the presence of the beta phase adjacent to the alpha phase boundaries and between α planes (Fig. 3). Thus, strain hardening during deformation at the lower temperature of 800 °C can be attributed to the pile-up and reaction of dislocations at the phase boundaries and within the alpha phase, respectively [41]. The increase in the beta phase fraction due to the rise in the test temperature can significantly result in strain rate distribution to the phases. During the initial stages of deformation, the beta phase with a lower hardness endures higher strain values. Therefore, it can be subjected to work hardening and increase the strength similar to the alpha phase. Accordingly, a high fraction of the alpha phase endures the applied stress. As a result, both alpha and beta β phases compete and are involved in the work-hardening at 885 °C [42]. On the contrary, the softening mechanism moderates the strain-hardening rate in strain values higher than 0.1. This implies that DRX or DRV phenomena activate at this stage (strain> 0.1) [43]. An analogous flow softening phenomenon was reported for Zr-Nb alloys [30] and Ti-6Al-4V alloy [44] within the alpha-beta region.

**Fig. 7:**
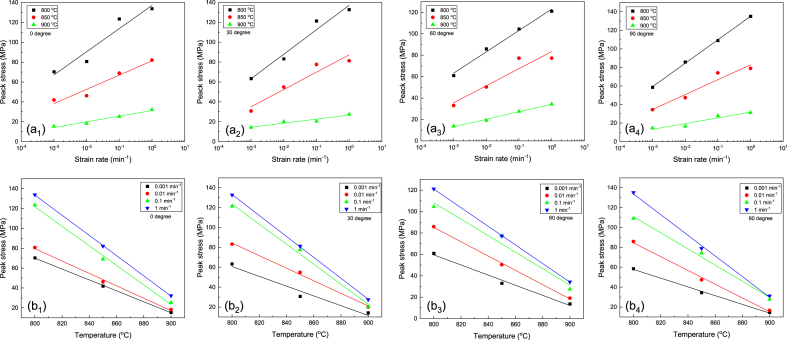
(a) Variation of peak stress vs strain rate in different directions, (b) Variation of peak stress vs temperature in different directions.

Fig. 7a depicts the diagram of peak stress variation in terms of strain rate for various temperatures. As shown in the figure, the maximum stress declines by reducing the strain rate. In other words, a longer time is provided for the interaction and annihilation of opposite-sign dislocations by reducing the strain rate. Likewise, when the time required for the grain nucleation for recrystallization is provided, the maximum stress is declined. Consequently, the temperature and strain rate are the parameters that influence the maximum stress, providing the conditions for DRX [45].

Fig. 7b reveals the changes in peak stress in terms of temperature for the compressed samples. According to the diagram, the value of peak stress declines by elevating the temperature at a constant strain rate. It is also apparent that the lowest peak stress is related to the sample compressed at 900 °C. Since the temperature is high enough to activate irregular sliding systems, cross slip reduces the dislocation density. Consequently, the DRV is activated by decreasing the high dislocation density, resulting in a decrease in the peak stress. Instead, the elevated temperature enhances the mobility of grain boundaries, accelerating the nucleation of recrystallized grains. Also, the low-angle grain boundaries formed during the previous stages convert into the primary boundaries, creating a microstructure consisting of dislocation-free grains and reducing the peak stress [46].

In this study, the constitutive equations were used to correlate the deformation parameters. To this end, the following Arrhenius equation (Equation (1)) was implemented to calculate the dependence of strain rate on the variations of stress and temperature [47].

(1) $$\dot{\varepsilon }=A{\left[\sinh\left(\alpha \sigma \right)\right]}^{n}\;\exp\left(-\frac{Q}{RT}\right)$$

where, α, n, and A and are material constant values, Q is the activation energy, R is the universal gas constant, T is the temperature [K]. If flow stress is high (i.e. ασ>0.8) or low (i.e. ασ<0.8), Equation (1) can be simplified as Equations (2), (3)).

(2) $$\dot{\varepsilon }=A_{1}\sigma^{n_{1}}\;\exp\left(-\frac{Q}{RT}\right)$$

(3) $$\dot{\varepsilon }=A_{2}\left(\beta \sigma \right)\exp\left(-\frac{Q}{RT}\right)$$

Equations (4), (5)) can be obtained by calculating the logarithm for both sides.

(4) $$\ln\dot{\varepsilon }={\ln\;A}_{1}+n_{1}\;\ln\;\sigma -\frac{Q}{RT}$$

(5) $$\ln\dot{\varepsilon }={\ln\;A}_{2}+\beta \sigma -\frac{Q}{RT}$$

**Fig. 8:**
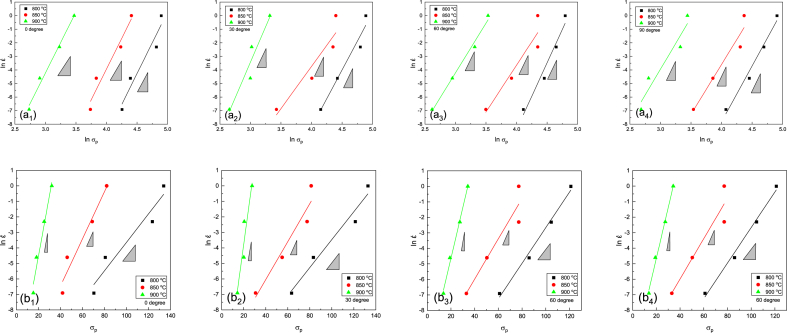
(a) Diagram of log strain rate vs log stress and (b) Diagram of log strain rate vs stress. (a_1_) and (b_1_) were reprinted from Ref. [42] with permission from Springer.

The logarithmic diagram of strain rate-stress is brought in Fig. 8a. By fitting a linear function on the graph, the value of n1 can be determined. The slope of this line denotes the n1 value. The average n1 values obtained were calculated as 9.04, 8.39, 8, and 7.71 for directions of 0, 30, 60, and 90° at different temperatures, respectively. As per Equation (5), by fitting a linear function on the logarithmic diagram of Fig. 8b, the β value was calculated from the experimental data. By calculating the average of the β values obtained at different temperatures, it was determined as 0.21, 0.24, 0.18, and 0.18 for the directions of 0, 30, 60, and 90°, respectively (Table 2).

**Table 2:** The calculated values for β at different temperatures.

Angle	Temperature (°C)		
	800	850	900
0	0.09	0.15	0.39
30	0.09	0.12	0.52
60	0.11	0.13	0.32
90	0.09	0.13	0.34

The α value was calculated by dividing β by n_1_ via Equation (6), which was 0.02 MPa^−1^ for all directions.

(6) $$\alpha =\frac{\beta }{n_{1}}$$

The activation energy was determined from Equation (7), via keeping the $\varepsilon \dot{\;}$ value constant.

(7) $$Q=R\;{\left\{\frac{\partial \ln\dot{\varepsilon }}{\partial \ln\left[\sinh\left(\alpha \sigma \right)\right]}\right\}}_{T}{\left\{\frac{\partial \ln\left[\sinh\left(\alpha \sigma \right)\right]}{\partial \left(\frac{1}{T}\right)}\right\}}_{\dot{\varepsilon }}$$

**Fig. 9:**
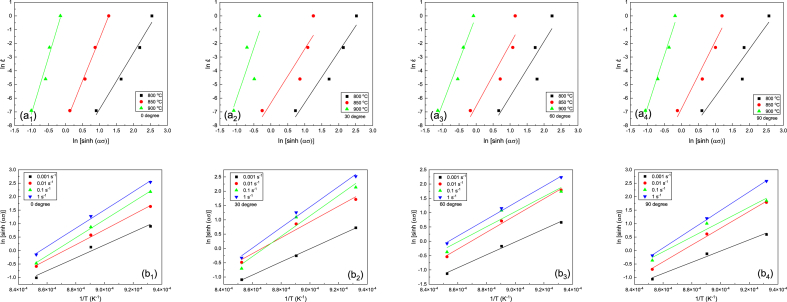
(a) Variation of log strain rate vs log sinh (ασ) and (b) Variation of log sinh (ασ) vs 1/T. (a_1_) and (b_1_) were reprinted from Ref. [42] with permission from Springer.

The variation of $\ln\dot{\varepsilon }$ according to ln[sinh(ασ)] at constant temperature is expressed as parameter ‘N’ is depicted in Fig. 9a to calculate the activation energy. If a linear function is fitted to the data, the line slope indicates parameter N. Similarly, the variation of ln[sinh(ασ)] vs 1/T at constant strain rate is expressed by symbol ‘S’ and is brought in Fig. 9b. Thus, the average values of activation energy were calculated as 304, 252, 223, and 305 kJ/mol using Equation (8) for the directions of 0, 30, 60, and 90°, respectively.

(8) Q=R×N×S

To determine the hot deformation mechanisms and construct the processing maps, n and Q values were used. The activation energy of Zr-1Nb alloy was calculated meaningfully higher than self-diffusion activation energies of Nb (132 kJ/mol), Zr (93 kJ/mol) [48], and O (130 kJ/mol) [49] in Zr-Nb alloys. In other words, Nb had a significant effect on enhancing the activation energy of the Zr-Nb series [50].

Moreover, the parameter of Zener–Hollomon (Z) was determined to assess the concurrent effects of strain rate and temperature on the flow stress [[51], [52], [53], [54]]. The relation between the parameter Z and flow stress is brought in Equation (9).

(9) $$Z=\dot{\varepsilon }\exp\left(\frac{Q}{RT}\right)=A{\left[\sin\left(\alpha \sigma \right)\right]}^{n}$$

where n is the stress constant. Furthermore, by deriving the logarithm of Equation (9), Equation (10) is resulted.

(10) lnZ=lnA+nln[sinh(ασ)]

Thus, the values of n were calculated as 5.96, 5.54, 6.24, and 5.87 for samples 0, 30, 60, and 90-degree from the slope and intercept of the fitted line, respectively. The n value can specify the mechanism of deformation. Once n > 5, cross slip is the dominant mechanism during deformation. While n values of 1, 2, and 3 imply that the dominant deformation mechanism is diffusional creep, grain boundary slip, and dislocations climb, respectively [31].

Generally, flow stress behavior at high temperatures is highly dependent on temperature and strain rate. The correlation between the flow stress and strain at constant temperatures complies with the power law [55]. The strain rate sensitivity (m) is determined from the hot-compression strain data via deriving natural logarithms of the power law (Equation (11)).

(11) $$\ln\;\sigma =\ln\;k+m\;\ln\dot{\varepsilon }$$

The maximum true stress is attained from the ln(*σ*P) polynomial expression according to ln($\dot{\varepsilon }$) (Equation (12)).

(12) $$\ln\;\sigma_{p}=a+b\;\ln\dot{\varepsilon }+c\;{\left(\ln\dot{\varepsilon }\right)}^{2}$$

where *a*, *b*, and c parameters are temperature-dependent,

In the case of steady flow, parameter m is calculated by deriving a natural logarithm of true stress-true strain data (Equation (13)).

(13) $$m=\frac{\partial \ln\;\sigma_{p}}{\partial \ln\dot{\varepsilon }}={\left[b+2c\;\ln\dot{\varepsilon }\right]}_{T.\varepsilon }$$

When the strain and temperature are constant, m is considered a function of strain rate and stress. The ratio of power dissipation efficiency during hot deformation is indicated below (Equation (14)).

(14) $$\eta =\frac{2m}{m+1}$$

The instability of the flow can occur during hot deformation, which can be determined if the below equation is fulfilled. According to Equation (15), if $\xi \left(\dot{\varepsilon }\right)<0$, the flow conditions are considered unsteady [56].

(15) $$\xi \left(\dot{\varepsilon }\right)=\frac{d\;\ln\left(\frac{m}{m+1}\right)\;}{d\;\ln\dot{\varepsilon }}+m=\frac{2c}{m\left(m+1\right)}+m\leq 0$$

Having the parameters of coefficient of power dissipation (η), strain rate sensitivity (m), and instability parameter (ξ) are necessary for the construction of processing maps. In addition, the microstructural changes are examined after the hot compression test to study the rheological instability.

**Fig. 10:**
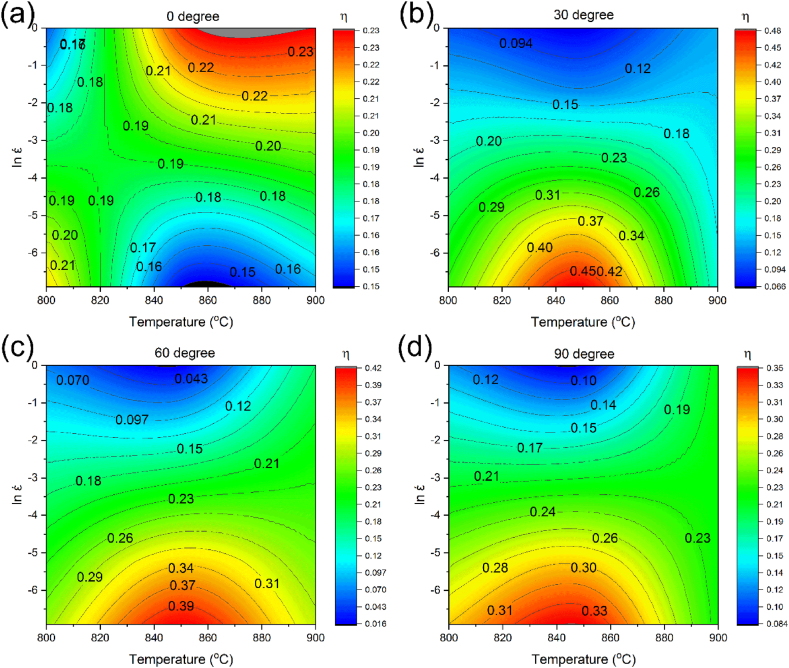
Diagram of power loss coefficient vs temperature and log strain rate in different forging directions: (a) 0° [42], (b) 30°, (c) 60°, and (d) 90°.

**Fig. 11:**
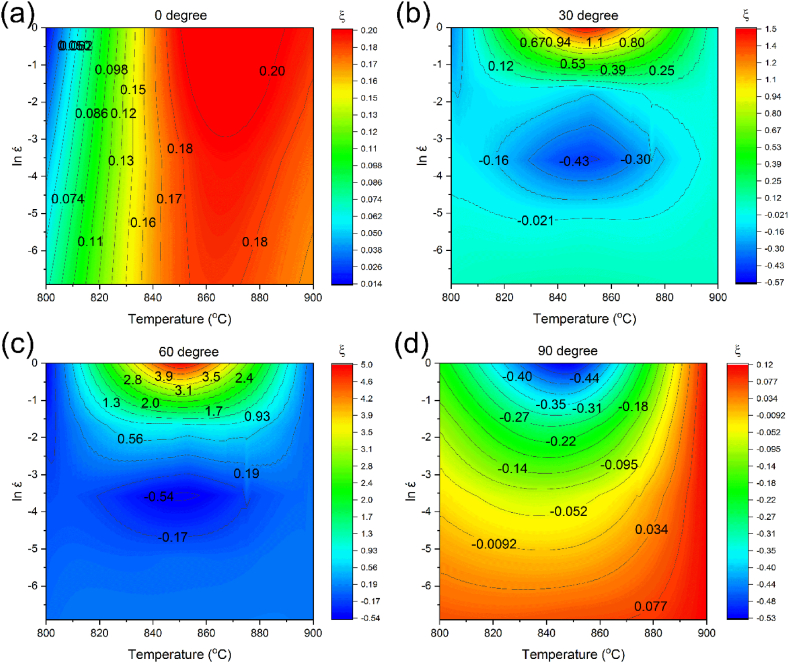
Variation of instability parameter vs temperature and log strain rate in different forging directions: (a) 0° [42], (b) 30°, (c) 60°, and (d) 90°.

Fig. 10a–d and 11a-d show the changes in η and ξ vs strain rate and temperature, respectively. Fig. 10 displays that sample zero-degree has a different behavior compared to the rest of the samples. For sample zero-degree, the power dissipation coefficient gradually increases with temperature elevation at high strain rates. Conversely, the power dissipation coefficient by increasing the temperature at low strain rates. For samples 30-, 60-, and 90-degree, the power dissipation coefficient at high strain rates stays nearly constant.

Moreover, at low strain rates, with increasing temperature, the power dissipation coefficient of the samples reaches a maximum and then decreases. Simultaneously, at low temperatures, the power dissipation coefficient of sample zero-degree first enhances and then reduces with increasing the strain rate. Eventually, at elevated temperatures, it reaches a maximum value. On the other hand, the power dissipation coefficients of samples 30, 60, and 90-degree decline with increasing the strain rate at a constant temperature. Although in general η indicates the material processability, the processing performance may not improve by increasing η. Consequently, within the processing instability region, the power dissipation coefficient can be high. As can be seen in Fig. 11; by elevating the temperature and strain rate, the instability parameter has enhanced slowly for samples zero and 90-degree. The trends of the samples 30 and 60-degree are almost similar, and their instability values at high strain rates first increase and then decrease with increasing temperature. Consequently, since the alloy deformation mechanism is complicated, determination of the instability and stability zones is necessary.

**Fig. 12:**
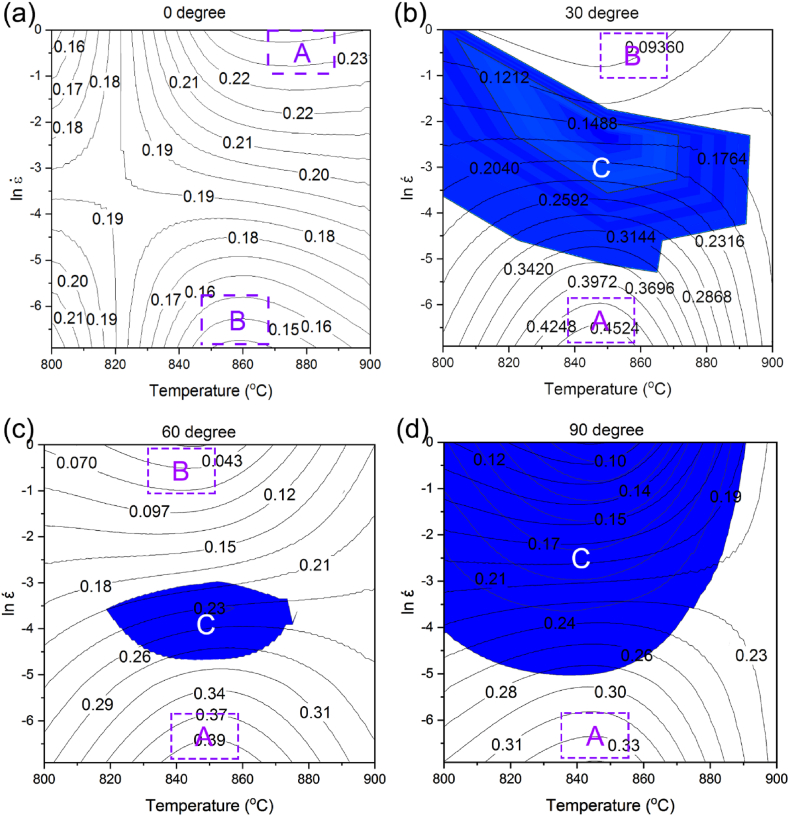
Hot processing map of the alloy in the temperature range of 800–900 °C and strain rate range of 0.001–1 s^−1^ in different forging directions: (a) 0° [42], (b) 30°, (c) 60°, and (d) 90°.

The hot processing map was attained by superposing the instability and power dissipation maps in the strain rate and temperature ranges of 0.001–1 s^−1^ and 800–900 °C, respectively (Fig. 12a–d). The contour lines indicate η, while different areas of ξ are shown in blue. As can be seen, the sample zero-degree has no instability zone within the ranges. While the power dissipation coefficient is high in areas A and B, the whole map is suitable for processing and there is no instability area. For samples 30 and 60-degree, the instability region is highlighted as blue within region C (Fig. 12b and c), which is an unsuitable region for processing. According to the map, the coefficient of power dissipation is high in region A, and it has no instability area. There is no instability area in zone B and the dissipation coefficient is low in the zone. Moreover, the properties of the deformed material are not as satisfactory as those of zone B. Therefore, zone A provides a superior processing area. As can be seen, sample 90-degree has a wide instability area, and similar to samples 30 and 60-degree, its highest power dissipation coefficient is in zone A.

As indicated in Fig. 12, the power dissipation negative values indicate that the zone of instability is located in the range of 800–890 °C, and the strain rates higher than 0.006 s^−1^ for samples 30-degree and 90-degree. For sample 60-degree, it is located in the range of 820–880 °C and the strain rate values of 0.006–0.049 s^−1^.

**Fig. 13:**
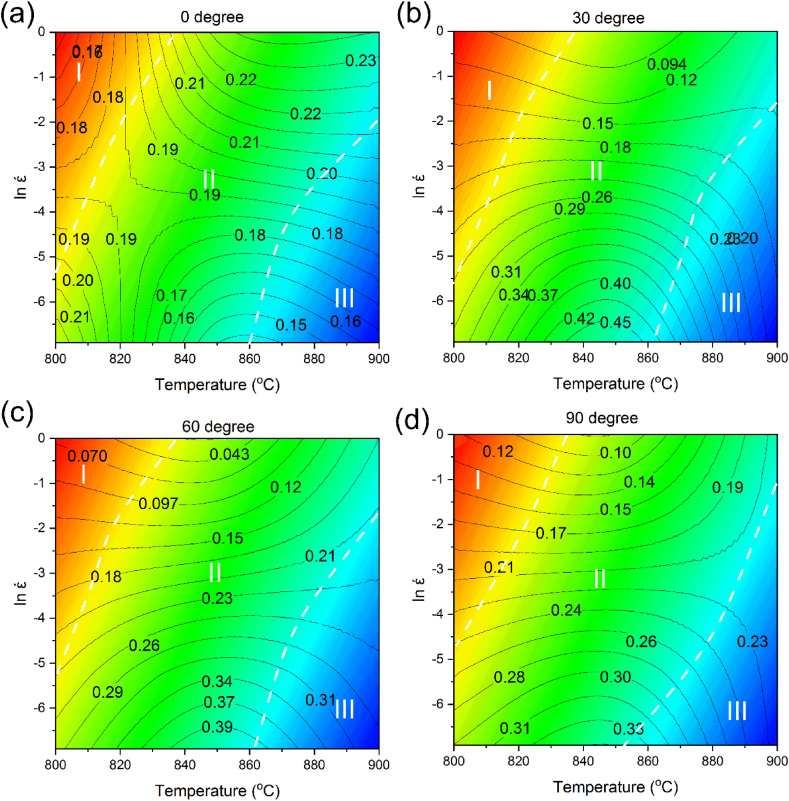
Superposing the Z parameter diagram on hot processing maps of Zr-1Nb in the temperature range of 800–900 °C and strain rate range of 0.001–1 s^−1^ in different forging directions: (a) 0° [42], (b) 30°, (c) 60°, and (d) 90°.

Parameter Z is used to determine the relation between strain rate, temperature, and flow stress. Numerous studies reported that Z can indicate the mechanism of softening [57,58]. Fig. 13a–d displays the superpose of the hot processing and parameter Z maps in the studied ranges. The contour lines and highlighted areas on the map represent the power dissipation coefficient and parameter Z, respectively. According to the figure, it is apparent that for all samples the behavior of parameter Z is similar. According to the figure the lowest Z value is located near 900 °C at 0.001 s^−1^. When ln (Z) is low, DRX and grain nucleation mechanisms are activated. On the other hand, the highest Z value is at 1 s^−1^ near 800 °C. This can be caused by unsatisfactory DRX as a result of a high strain rate with increasing strain. Furthermore, when the Z value is high, the rearrangement of dislocations improves, which accelerates the DRV phenomenon. It is evident that the Z value steadily declines by passing from domain I to III. This suggests that the DRV mechanism (when Z is high) converts to the DRX mechanism (with low Z values) through softening. Thus, it was deduced that the hot deformation conditions of the alloy were improved by changing the dominant mechanism of DRV to DRX. Moreover, DRX is satisfactory during the processing since it encourages steady rheology. The area is frequently favored for control of microstructure and process optimization.

**Fig. 14:**
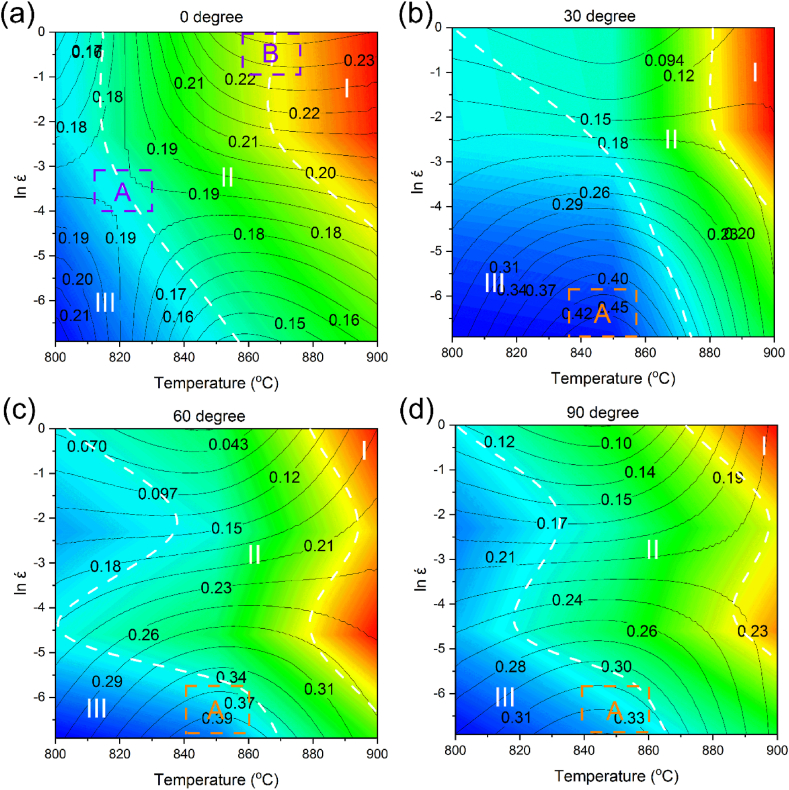
Superposing the activation energy diagram on hot processing maps of the alloy in the temperature range of 800–900 °C and strain rate range of 0.001–1 s^−1^ in different forging directions: (a) 0°, (b) 30°, (c) 60°, and (d) 90°.

Fig. 14a–d shows the superposing of the hot processing map and activation energy graphs within the studied ranges. The contour lines in Fig. 14 represent the power dissipation efficiency value. The activation energy values are highlighted in color on the graph. According to the figure, the highest activation energy is attained at 900 °C and high strain rates. High Q values imply more difficult hot processability, indicating the need for optimizing the optimal area of processing even further. For sample zero-degree, Q is low and η has its maximum value in region A. In region B, Q is moderate, but the contour lines are closely distributed. This highlights that minor changes in the process conditions can highly affect the Q value, therefore this range is not suitable for hot deformation [59,60]. Area B has a higher Q value than that of area A, nonetheless, the contour lines are scattered in area A. The stability of deformation in region B is typically attributed to DRX. The trend of Q and η is similar for samples 30, 60, and 90-degree. In area A, Q is low, while η has its highest value.

### 3.6 Microstructure characterization after hot compression

**Fig. 15:**
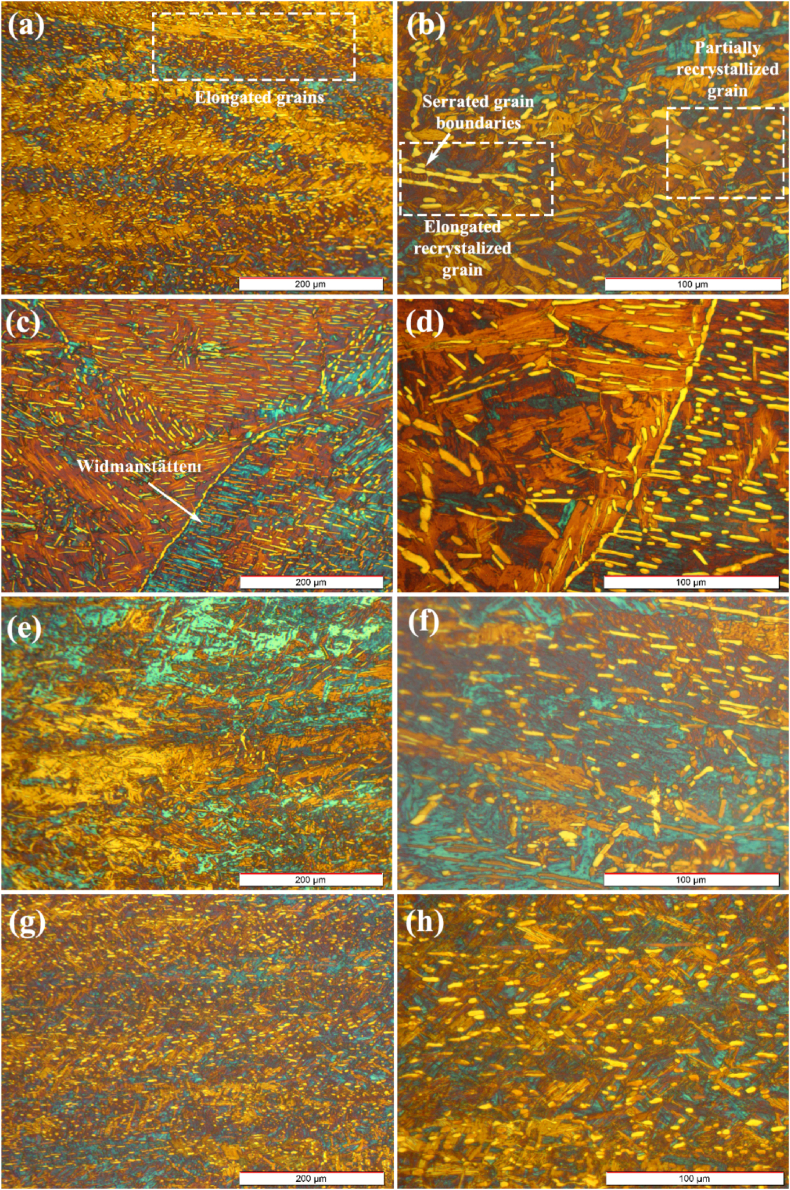
Optical micrographs of the forged alloy after compression test at 800 °C and a strain rate of 0.001 s^−1^ with different magnifications in directions of: (a, b) 0°, (c, d) 30°, (e, f) 60°, and (g, h) 90°.

Fig. 15a and b shows the microstructure of the sample zero-degree following the hot compression test at 0.001 s^−1^ and 800 °C. As it is apparent in Fig. 15b, the microstructure consists of serrated grain boundaries around elongated grains, which are attributed to deformation at low temperatures (bottom of the two-phase region) and low strain rate. A similar microstructure has been reported by researchers during hot compression [61]. However, in some areas, the refined and recrystallized alpha phase has been dispersed within the primary alpha phase, which is in some cases elongated vertically along the deformation direction. In other cases, they are spherical. This is attributed to the sufficient time provided for directional grain growth due to the low strain rate. By relating the stress-strain diagrams to the microstructures of the sample microstructures, it is apparent that DRX and DRV mechanisms play an important role in softening. Thus, at higher temperatures, both mechanisms dominate gradually.

Fig. 15c and d demonstrate the optical micrograph of sample 30-degree following the hot compression at 0.001 s^−1^ and 800 °C. Low-magnification micrograph shows that the recrystallized α phase is homogeneously distributed in the microstructure, which is mostly elongated in the same direction. The orientation of the recrystallized grains is more evident near the grain boundaries, resulting in the nucleation of parallel planes at the grain boundaries, which grow into the grains. A previous study reported that this type of microstructure had less processability [62]. Moreover, changes in localized microstructural (parallel laths) are apparent adjacent to the grain boundaries. In the case of zirconium alloys, this microstructure represents Widmanstätten basket weave, which is more probable at low cooling rates in the β region.

Fig. 15e and f shows the optical micrograph of sample 60-degree following the hot compression at 0.001 s^−1^ and 800 °C. It can be seen that the microstructure has transformed into a plane where the interlaced α-structure is surrounded by the grain boundaries of the primary β-grains. The interlaced α planes form a large number of interfaces, indicating that the sample has a high interface energy. The microstructure evolved from the high driving force caused by the applied load during the test and the previous internal strains induced by the forging process. The driving force is released during the hot compression in the form of diffusion and the recrystallization processes [63,64]. The distribution of recrystallized α phase in the micrograph with higher magnification shows that the fraction of recrystallized phase in sample 60-degree is lower than those of samples zero and 60-degree. Fig. 15g and h reveal the micrograph of sample 90-degree following the hot compression at 800 °C and 0.001 s^−1^. As can be seen, the homogenous distribution of recrystallized fine-grained α-phase is dispersed throughout the sample, which is more spherical and less elongated than that of sample zero-degree.

**Fig. 16:**
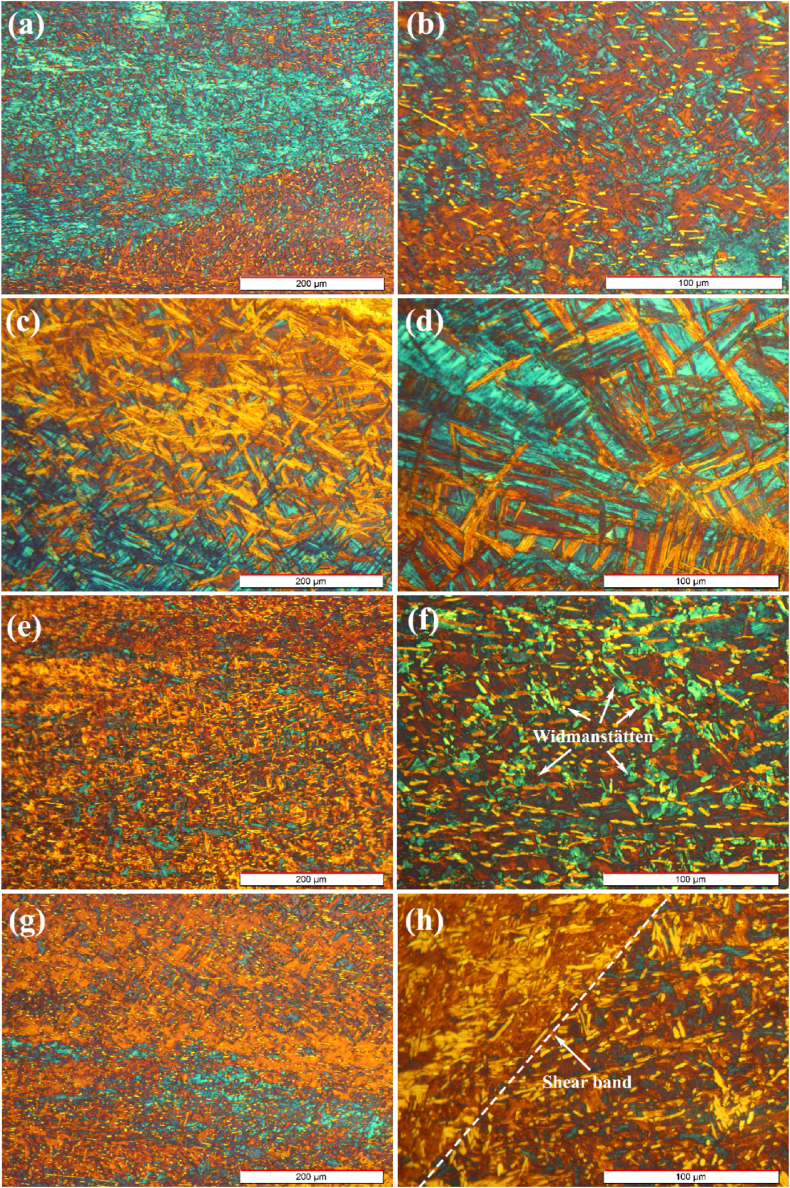
Optical micrographs of the forged alloy after compression test at 800 °C and a strain rate of 1 s^−1^ with different magnifications in directions of: (a, b) 0°, (c, d) 30°, (e, f) 60°, and (g, h) 90°.

Fig. 16a and b shows the microstructure of sample zero-degree after the hot compression at 1 s^−1^ and 800 °C. It can be seen that the recrystallized refined alpha phase is distributed homogenously within the microstructure. A comparison of Fig. 16, Fig. 15b shows that the new recrystallized phases nucleated at 1 s^−1^ are significantly finer than those nucleated at 0.001 s^−1^. This can be related to the inadequate time needed for grain growth and diffusion. Fig. 16c and d displays the microstructure of sample 30-degree hot-compressed at 1 s^−1^ and 800 °C. Several martensite laths with different widths are observed in the zirconium alloy matrix in the size of several hundred nanometers. The pile-up of the dislocations due to hot-working provides sites preferable for transformation nucleation and promotes martensite lath formation. Depending on the cooling rate, α-platelets are formed by strong epitaxy on primary β-grains [65,66], causing the alloying elements to be completely dissolved in the β-phase which significantly promotes grain growth. After cooling, β-grains transform into α-needles by rejecting elements, which stabilize the β-phase to the grain boundaries. Furthermore, the formation of relatively fine basketweave microstructure within the α phase can be due to the existence of numerous nucleation sites inside the primary β matrix. Moreover, it can be said that these basketweave plates intersect each other within the primary β matrix. Another morphology is parallel plates, consisting of numerous long plates that grow from β-phase boundaries [67]. However, the difference is attributed to secondary phase precipitates that are dispersed randomly, causing the nucleation of α phase planes and producing a basket weave structure. Fig. 16e and f shows the optical micrograph of sample 60-degree after the hot compression at 800 °C and s^−1^. According to the micrograph, the Widmanstätten structure is scattered in the alloy matrix. Also, recrystallized α phases are distributed in the microstructure. Fig. 16g and h demonstrate the optical micrograph of sample 90-degree hot-compressed at 1 s^−1^ and 800 °C. The morphology of the shear bands reveals nonuniform recrystallization. Adiabatic shear bands appear at high strain rates in the microstructure, when the generated deformation heat cannot be transferred caused by the limited thermal conductivity of Zr-1Nb alloy [49,68,69].

**Fig. 17:**
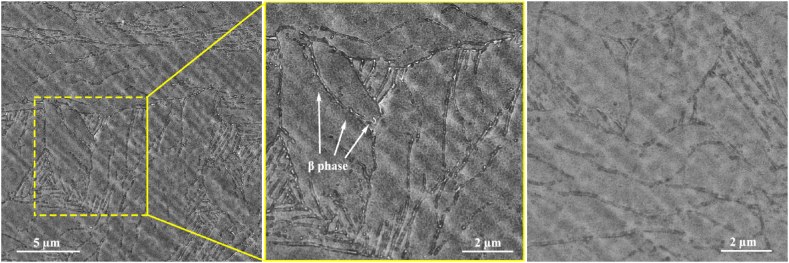
SEM micrographs of the forged alloy after compression test at 800 °C in direction of 90° with the strain rate of 1 s^−1^.

SEM micrograph was utilized to further investigate the microstructure in the initial as-forged state (Fig. 17). As it is evident, a skeletal structure of the secondary phase participates at the boundaries was formed. Moreover, it can be seen that thinner laths were attained in this investigation in comparison to previous ones [62]. A recent investigation [70] stated that the width of laths is related to the oxygen concentration and cooling rate of the alloy, i.e. in case of prolonged holding time, the oxygen content can be increased. This results in an increase in the martensite start temperature, which expands the α+β region. With prolonging the holding time, oxygen diffusion from β to α phases is enhanced. Thus, diffusion-controlled phase transformation improves, which increases the martensite lath width. Based on the figure, there are numerous lath regions and coarse martensite plates in the microstructure. The formation of the lath regions, which are the same size and orientated with a 60° angle together implies the favored direction for martensite lath growth. These results are similar to the microstructure of Zr–Sn–Nb–Fe alloy quenched from the β-region in water presented by Chai et al. [71]. Also, it has been found that the size of the plate and acicular martensite phases are hardly affected by the test temperature. Moreover, the diffusion-controlled phase transformations are encouraged by hot compression. In other words, the diffusion of atoms that cannot transform between alpha laths due to insufficient time is enhanced within the beta phase and α-lath boundaries.

**Fig. 18:**
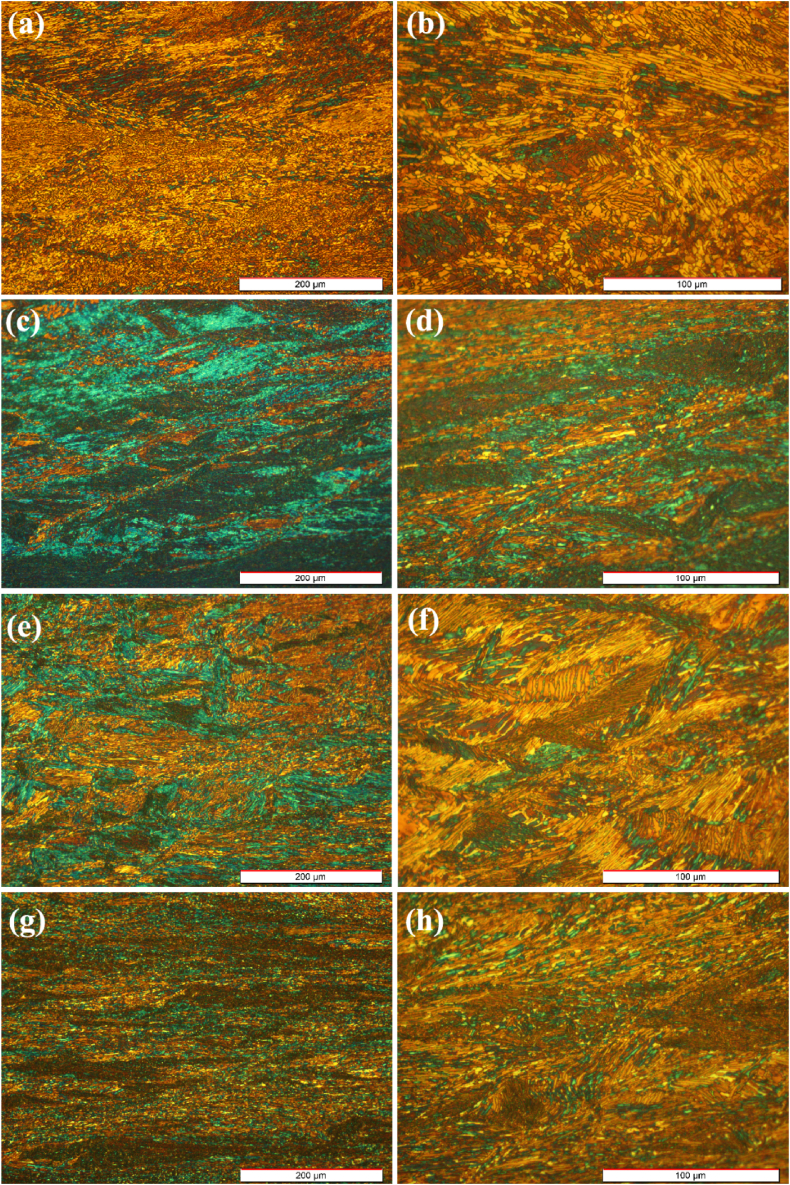
Optical micrographs of the forged alloy after compression test at 850 °C in direction of 60° with a strain rate of: (a, b) 0.001 s^−1^, (c, d) 0.01 s^−1^, (e, f) 0.1 s^−1^, and (g, h) 1 s^−1^.

Fig. 18 shows the microstructure of sample zero-degree hot-compressed at 850 °C and different strain rates. As can be realized, equiaxed and recrystallized α-Zr grains have formed at 0.001 s^−1^, which means DRX is dominant (Fig. 18a and b). At 0.01 s^−1^, partial recrystallization along with a small portion of recrystallized fine grains of α-Zr can be detected. Thus, the dominant mechanism of softening was DRV during the deformation at 0.01 s^−1^ and 850 °C (Fig. 18c and d). Flow localization areas are apparent at 0.1 and 1 s^−1^ (Fig. 18e–h). In the two-phase alpha-beta region (particularly 800–900 °C), the beta-Zr fraction is highly enhanced compared to that of the alpha-Zr phase. Consequently, by increasing the test temperature to 850 °C, the required stress for DRV was reduced to nearly 30 % at all strain rates. The hot-deformed microstructure displays and confirms the flow localization at 850 °C and 1 s^−1^.

## 4 Conclusions

The present study evaluated the processing map and microstructural evolution of Zr-1Nb alloy in different directions after bidirectional hot forging. To this purpose, zirconium ingots containing 1 % niobium were cast using the vacuum arc remelting process. The ingot was preheated and forged by the isothermal bidirectional free forging process. The microstructural evolution and hardness variation are as follows.

1) The as-cast microstructure consisted of allotriomorphic alpha phases with different morphologies, which were located inside beta grains and beta grain boundaries.
2) Sample 90-degree had the highest fraction of elongated α grains as a result of the high strain induced by forging. This resulted in the formation of new refined α along the direction of forging.
3) The hardness measurement results showed that the highest value was related to sample 90-degree due to the high fraction of the α phase, its morphology, and refinement during the forging process. By comparing the hardness of the as-cast sample with the forged samples, it is evident that the hardness of forged samples increased by 20 %, 5 %, 12 %, and 35 % for samples 0, 30, 60, and 90-degrees, respectively.
4) The mechanisms and kinetics of DRX varied for samples having different crystalline orientations and were dependent on the activated deformation mechanisms. Sample 90-degree showed a high resistance to DDRX. Thus, basal and pyramidal ⟨a⟩ slip mechanisms were dominant at the initial deformation stage. Additionally, the strain hardening rate of sample 90-degree was lower than that of sample zero-degree. The dominant CDRX increased with grain rotation. Hence, it can be said that the texture-induced softening at the initial deformation stage masked DDRX-induced softening.
5) The stress-strain diagram trends and optical micrographs implied that DRX was the key mechanism of deformation, which led to grain refinement.
6) The optimum deformation area in the processing map was recognized within the ranges of 0.001–0.01 s^−1^ and 800–900 °C.
7) When the strain rate was higher than 0.1 s^−1^, instability was detected as adiabatic shear bands and flow localization. The strain hardening exponents for samples 0, 30, 60, and 90-degree were determined as 5.96, 5.54, 6.24, and 5.87, respectively. Moreover, the activation energy was calculated as Q ∼304 kJ/mol.

## CRediT authorship contribution statement

**Ali Rajaee:** Writing – original draft, Methodology, Investigation, Conceptualization. **Mohsen Asadi Asadabad:** Writing – review & editing, Methodology, Conceptualization. **Behrooz Shayegh Boroujeny:** Methodology, Conceptualization.

## Data availability

Data will be made available on request.

## Declaration of competing interest

The authors declare that they have no known competing financial interests or personal relationships that could have appeared to influence the work reported in this paper.
